# Mechanisms of relapse in acute leukaemia: involvement of p53 mutated subclones in disease progression in acute lymphoblastic leukaemia

**DOI:** 10.1038/sj.bjc.6690183

**Published:** 1999-03

**Authors:** Y-M Zhu, L Foroni, I G McQuaker, M Papaioannou, A Haynes, H H Russell

**Affiliations:** Department of Haematology, Nottingham City Hospital, Nottingham, N65 1PB, and the University of Nottingham, UK; Department of Haematology, Royal Free Hospital and School of Medicine, London, UK

**Keywords:** p53, mutations, acute lymphoblastic leukaemia, acute myeloblastic leukaemia, clonal selection

## Abstract

Mutations of the *p53* tumour suppressor gene are infrequent at presentation of both acute myeloblastic leukaemia (AML) and acute lymphoblastic leukaemia (ALL), being found in between 5–10% of AML and 2–3% of ALL. Here we have studied the frequency of detection of p53 mutations at relapse of both AML and B-precursor ALL. In those patients with detectable mutations at relapse we investigated whether the mutation was detectable at presentation and was thus an early initiating event or whether it had arisen as a late event associated with relapse. Bone marrow samples from 55 adults and children with relapsed AML (*n* = 41) or ALL (*n* = 14) were analysed for *p53* gene alterations by direct sequencing of exons 5–9. For samples where a *p53* mutation was found at relapse, analysis of presentation samples was carried out by direct sequencing of the exon involved, or by allele-specific polymerase chain reaction (PCR) if the mutation could not be detected using direct sequencing. A *p53* mutated gene was found at relapse in seven out of 55 cases. The frequency was higher in relapsed ALL (four out of 14 cases; 28.6%) compared to AML (three out of 41 cases; 7.3%). In five out of the seven cases presentation samples were available to study for the presence of the mutation. In two out of two AML patients the *p53* mutation was detectable in the presentation sample by direct sequencing. In three ALL patients analysis of presentation material by direct sequencing showed a small mutant peak in one case, the other two being negative despite the sample analysed containing > 90% blast cells. However in both of these patients, the presence of *p53* mutation was confirmed in the presentation sample using allele-specific PCR. In one of these patients the emergence of a subclone at relapse was confirmed by clonality analysis using IgH fingerprinting. Our results confirm that in ALL *p53* mutations are present in a proportion of patients at relapse. Furthermore cells carrying the mutation are detectable at presentation in a minor clone suggesting that *p53* mutations in ALL may be a mechanism contributing to disease relapse. © 1999 Cancer Research Campaign


*British Journal of Cancer* (1999) **79**(7/8), 1151–1157
© 1999 Cancer Research Campaign
Article no. bjoc.1998.0183

### Mechanisms of relapse in acute leukaemia: involvement of p53 mutated subclones in disease progression in acute lymphoblastic leukaemia

**Y-M Zhu[1], L Foroni[2], IG McQuaker[1], M Papaioannou[2], A Haynes[1] and HH Russell[1]**

[1]Department of Haematology, Nottingham City Hospital, Hucknall Road, Nottingham N65 1PB, and the University of Nottingham, UK; [2]Department of Haematology, Royal Free Hospital and School of Medicine, London, UK

**Summary** Mutations of the *p53* tumour suppressor gene are infrequent at presentation of both acute myeloblastic leukaemia (AML) and acute lymphoblastic leukaemia (ALL), being found in between 5–10% of AML and 2–3% of ALL. Here we have studied the frequency of detection of p53 mutations at relapse of both AML and B-precursor ALL. In those patients with detectable mutations at relapse we investigated whether the mutation was detectable at presentation and was thus an early initiating event or whether it had arisen as a late event associated with relapse. Bone marrow samples from 55 adults and children with relapsed AML ($n$ = 41) or ALL ($n$ = 14) were analysed for *p53* gene alterations by direct sequencing of exons 5–9. For samples where a *p53* mutation was found at relapse, analysis of presentation samples was carried out by direct sequencing of the exon involved, or by allele-specific polymerase chain reaction (PCR) if the mutation could not be detected using direct sequencing. A *p53* mutated gene was found at relapse in seven out of 55 cases. The frequency was higher in relapsed ALL (four out of 14 cases; 28.6%) compared to AML (three out of 41 cases; 7.3%). In five out of the seven cases presentation samples were available to study for the presence of the mutation. In two out of two AML patients the *p53* mutation was detectable in the presentation sample by direct sequencing. In three ALL patients analysis of presentation material by direct sequencing showed a small mutant peak in one case, the other two being negative despite the sample analysed containing > 90% blast cells. However in both of these patients, the presence of *p53* mutation was confirmed in the presentation sample using allele-specific PCR. In one of these patients the emergence of a subclone at relapse was confirmed by clonality analysis using IgH fingerprinting. Our results confirm that in ALL *p53* mutations are present in a proportion of patients at relapse. Furthermore cells carrying the mutation are detectable at presentation in a minor clone suggesting that *p53* mutations in ALL may be a mechanism contributing to disease relapse.

**Keywords:** p53; mutations; acute lymphoblastic leukaemia; acute myeloblastic leukaemia; clonal selection

*p53* has been shown to be one of the most frequently mutated genes in human cancers (Lane and Benchimol, 1990; Vogelstein, 1990). Several studies have shown that mutations of the *p53* gene in both acute myeloblastic leukaemia (AML) and acute lymphoblastic leukaemia (ALL) are relatively infrequent in contrast to solid tumours where point mutations are common. Although *p53* mutations are common in AML cell lines (Sugimoto et al, 1992), the reported frequency in primary AML cases is much lower (5–10%) (Slingerland et al, 1991; Fenaux et al, 1992*a*; Hu et al, 1992). The incidence of *p53* mutations in AML is higher with presence of monosomy 17p (Fenaux et al, 1991). In addition, AML arising secondary to previous chemotherapy has a high incidence of *p53* mutations (Ben-Yehuda et al, 1996). In ALL, a low incidence of *p53* mutations has been reported (Wada et al, 1993): thus in a large study of 330 patients with paediatric ALL at diagnosis only eight patients with mutations were detected. These observations have led to the suggestion that *p53* mutations are of less significance in acute leukaemia compared to other tumours. However, evidence to the contrary has come from a number of reports. Firstly AML associated with *p53* mutations has a very poor prognosis with a low complete remission rate and a poor survival (Wattel et al, 1994). Secondly in T-ALL the incidence of p53 mutations was found to occur at a much higher frequency in leukaemic cells at relapse (24% of cases) compared to patients studied at presentation where *p53* mutations are rare (Jonveaux et al, 1991; Mori et al, 1992; Diccianni et al, 1994; Hsiao et al, 1994). These latter findings could be interpreted as indicating that *p53* mutations are associated with clonal evolution in acute leukaemia and are therefore a 'late' event, as in chronic myeloid leukaemia (Nakai et al, 1993). An alternative explanation for the findings in T-ALL is that the mutation is present at presentation but only in a minor subclone which is able to survive remission induction and consolidation chemotherapy, mediating disease relapse. This possibility is supported by data from Wada et al (1994) who identified *p53* mutations in cell lines established from patients with relapsed leukaemia. In none of the cases could they identify the mutation in presentation samples using single-strand conformation polymorphism (SSCP) analysis. However, using the more sensitive technique of allele-specific PCR they were able to identify a mutated subpopulation accounting for between 0.01% and 1% of presentation cells.

To gain further insight into the role of *p53* mutation in the evolution of acute leukaemia, we have studied the structure of the

*Received 20 March 1998*
*Revised 30 June 1998*
*Accepted 25 September 1998*

*Correspondence to:* NH Russell

**Table 1** Clinical characteristics of patients with *p53* mutations at relapse

| Patients | Diagnosis (FAB type) | Sex/age | Cytogenetics | Mutated *p53* gene | | | |
|---|---|---|---|---|---|---|---|
| | | | | Exon | Codon | Nucleotide change | Amino acid change |
| 1. BHM | c-ALL | M/3 | 46 XY, –4, –12p, +14, +21 | 5 | 175 | CGC→CAC | Arg→His |
| 2. GD | AML (M4) | F/58 | 47 XX, +8 | 8 | 273 | CGT→TGT | Arg→Cys |
| 3. IR | ALL (B) | F/25 | 46 XX, t(8;14) | 6 | 193 | CAT→CGT | His→Arg |
| 4. SR | AML (M1) | M/55 | 46 XY, –4q, –7q, –7, –21 | 7 | 241 | TCC→TGC | Ser→Cys |
| 5. WG | ALL (pre-B) | F/17 | 47 X, –X, –4, –6(q2), +13, +13, +14, –20 +mar | 6 | 209 | AGA→ACA | Arg→Thr |
| 6. WN | c-ALL | M/32 | 46 XY | 5 | 175 | CGC→TGC | Arg→Cys |
| 7. WS | AML (M6) | F/32 | 43 XX, –5, –7, –18, –22, +12p | 7 | 248 | CGG→TGG | Arg→Thr |

ALL, acute lymphoblastic leukaemia; AML, acute myeloblastic leukaemia.

*p53* gene in leukaemic cells from a series of patients at relapse by direct sequencing of exons 5–9. We detected a *p53* mutation in four out of 14 (28.6%) patients with ALL suggesting that mutations of *p53* are much more frequently detected in relapsed samples than at presentation. However, in all cases the mutation could be detected at presentation in a minority subclone either by direct sequencing or by allele-specific PCR.

### MATERIALS AND METHODS

### Patients

Bone marrow samples from 55 patients with relapsed acute leukaemia were analysed including 41 AML (median age 49 years; range 21–80 years) and 14 B-lineage ALL (median age 29 years; range 3–54 years) patients. Samples had been previously cryopreserved in 10% dimethyl sulphoxide (DMSO) in a controlled-rate freezer. All patients had achieved a complete remission having received treatment with intensive chemotherapy according to the Medical Research Council trials of AML-9 or AML-10 for patients with AML or UKALL-10, UKALLXa or UKALL-12 for patients with ALL.

### Primers for exon 5–9 of *p53*

Oligonucleotide primers for the PCR and sequence reactions were purchased from R&D Systems Europe Ltd (Abingdon, UK). The nucleotide sequences of the primers used for PCR of exons 5–9 were:

5S: 5′-CCTCTTCCTGCAGTAC-3′
6AS: 5′-AGTTGCAAACCAGACCTC-3′
7S: 5′-CTCCTAGGTTGGCTCTGAC-3′
7AS: 5′-CAAGTGGCTCCTGACCTGG-3′
8S: 5′-CCTATCCTGAGTAGTGG-3′
9AS: 5′-AAGACTTAGTACCTGAAGGGT-3′

The sizes of PCR products were: 406 bp for exon 5–6, 148 bp for exon 7 and 324 bp for exon 8–9.

### PCR and direct sequencing analysis

High molecular weight DNA was prepared from cryopreserved peripheral blood or bone marrow cells from patients at relapse. DNA was extracted by a DNA extraction kit (Stratagene, La Jolla, CA, USA) following the manufacturer's instructions.

PCR reaction was carried out in a 50 μl volume including 1 mM magnesium chloride, 0.12 mM of each dNTP, 1 × buffer (20 mM Tris-HCl pH 8.4, 50 mM potassium chloride), 200 ng of genomic DNA, 1 U of *Taq* polymerase (Promega, Madison, WI, USA) and 1 μM of both the upstream and downstream PCR primers. Amplification was carried out in Techne Thermal Cycler, PHC-3 after an initial denaturation at 94°C for 3 min. A total of 35 cycles were performed using the following temperature and time profile: denaturation at 94°C for 1 min; primer annealing at 55°C (for exon 5–6), 62°C (for exon 7) and 57°C (for exon 8–9) for 1 min; primer extension at 72°C for 1 min; and a final extension of 72°C for 5 min. The PCR products were purified by Chroma Spin™ Columns (Clontech, Palo Alto, CA, USA). The PCR products for exon 7 were purified by Chroma Spin-100. The PCR products for exon 5–6 and exon 8–9 were purified by Chroma Spin-200.

The PCR products were sequenced by both forward and reverse primers in each sample. About 80 ng of purified PCR products for exon 7, and 180 ng of purified PCR products for exon 5–6 and exon 8–9 were added to the sequencing reaction. The final reaction volumes were 20 μl also including 8.0 μl of Terminator Ready Reaction Mix (Perkin Elmer, Foster City, CA, USA), 3.2 pmol of forward or reverse primer and $dH_2O$. The cycle sequencing was performed for 25 cycles in PTC-100TM Programmable Thermal Controller (MJ Research Inc, Watertown, MA, USA) as following programme: 96°C for 30 s, 50°C for 15 s and 60°C for 4 min. The extension products were purified by an ethanol precipitation protocol. The dried pellets were dissolved in 6 μl of loading buffer by combining 5 μl of deionized formamide and 1 μl of 25 mM EDTA (pH 8.0) containing 50 mg $ml^{-1}$ of blue dextran. The samples were vortexed and heated to 90°C for 2 min to denature, then cooled on ice until ready to load. Samples (1.5 μl) were then loaded to a 4.2% polyacrylamide gel containing 8.3 M urea. The sequence analysis was performed on a 377 automated DNA sequencer (PE Applied Biosystems, Foster City, CA, USA). Where a mutation was detected this was confirmed on a second occasion by a separate PCR reaction and repeat sequencing.

### Demonstration of mutant p53 at presentation by allele-specific gene amplification

As the failure to detect *p53* mutations in presentation samples might reflect the lack of sensitivity of direct sequencing to detect

 © Cancer Research Campaign 1999

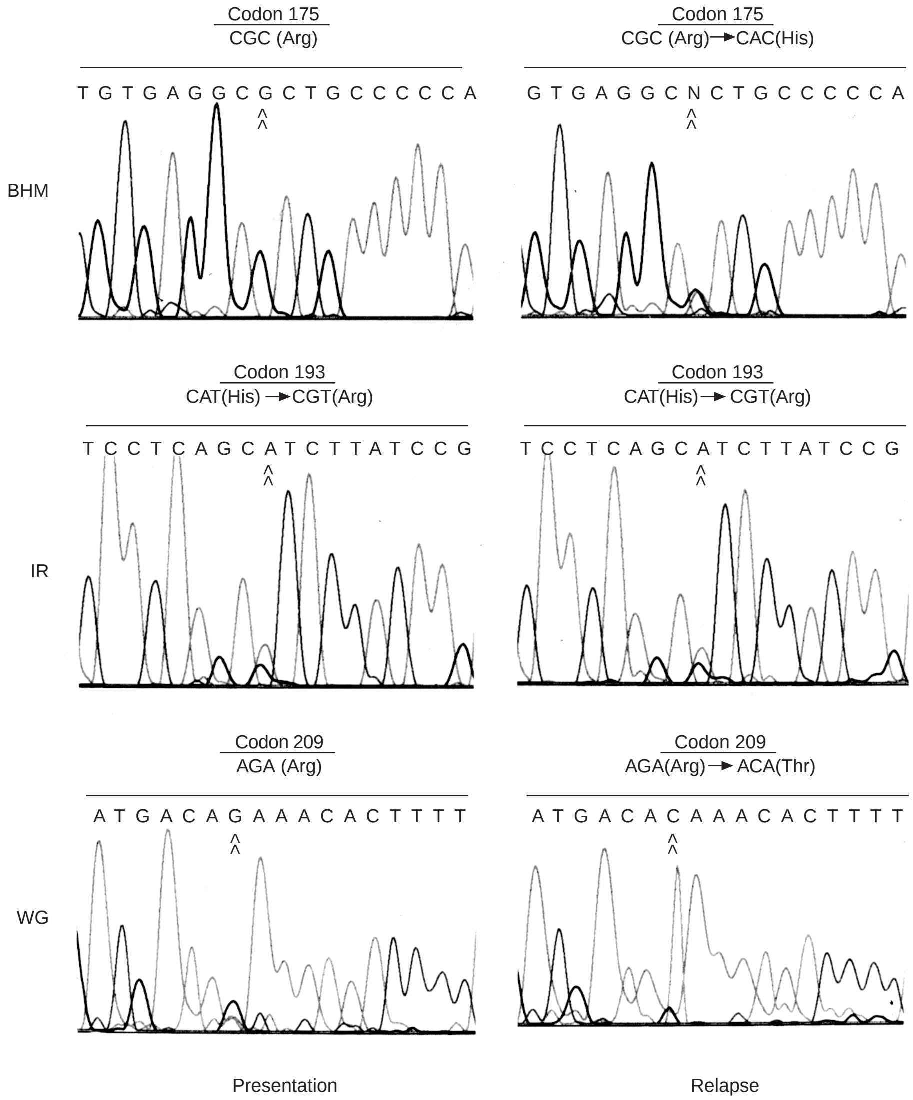

**Figure 1** Comparisons of *p53* mutations detected by direct sequencing at presentation (left) and at relapse (right) of three ALL patients: BHM (top), IR (middle) and WG (bottom). A CGC→CAC transition occurred in codon 175, resulted in Arg→His substitution (BHM). A CAT→CGT transition in codon 193, resulted in a His→Arg substitution in patient IR. An AGA→ACA transversion in codon 209, resulted in an Arg→Thr substitution in patient WG. Analysis of the presentation samples confirmed the presence of the mutation in IR, but not in BHM or WG

the presence of a minority clone, we proceeded to use allele-specific PCR to re-analyse the presentation sample in two patients (BHM and WG) where we had failed to detect the mutation by sequencing. The allele-specific primer was so designed that they differed from the normal allele in that their terminal 3′ nucleotide was underlined in the nucleotide sequence as following: 5′-ATGACGGAGGTTGTGAGGCA-3′ (for BHM); 5′-CACTATGTCGAAAAGTGTTTG-3′ (for WG). The conditions of the PCR reaction were similar to that described earlier except that the annealing temperature was 68°C (for BHM) and 65°C (for WG). The expected size of PCR product was 264 bp (for BHM) and 369 bp (for WG).

### IgH FR1 fingerprinting

The fingerprinting test was carried out in patient BHM according to a previously described method (Chim et al, 1996). Briefly, the FR1 primers (VH3, VH4a and VH4b) were used in combination with the JH primer in a 20 μl final volume of PCR reaction including α-$^{32}$p dCTP. After 25 cycles of PCR (94°C for 1 min, 54°C for 1 min and 72°C for 1 min each cycle), the products were denatured by boiling and loaded onto a sequencing acrylamide gel (6% acrylamide and 8 M urea). The gel was run at 30 W for 2.5 h at room temperature in 0.5 × TBE, then was transferred to a sheet of 3 MM Whatman paper, and dried at 80°C. Autoradiography was carried out at – 70°C for about 24 h with an intensifying screen.

### RESULTS

Of the 55 patients with relapsed acute leukaemia analysed by direct sequencing of exons 5–9, *p53* mutations were detected in a total of seven (12.7%). This consisted of four out of 14 (28.6%) patients with ALL but only three of 41 (7.1%) patients with AML. Clinical and cytogenetic data of the patients with *p53* mutation at

© Cancer Research Campaign 1999

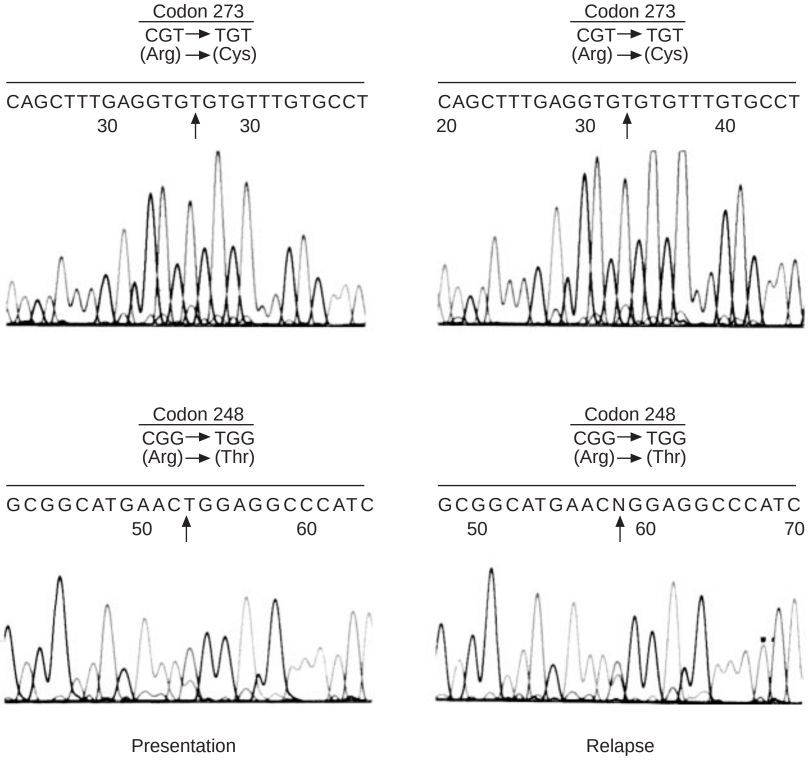

**Figure 2** Comparisons of *p53* mutations detected by direct sequencing at presentation (left) and at relapse (right) of two AML patients: GD (top) and WS (bottom). A CGT→TGT transition occurred in codon 273, resulted in Arg→Cys substitution (GD). A CGG→TGG transition in codon 248, resulted in an Arg→Thr substitution in patient WS. Analysis of the presentation samples confirmed the presence of the mutations in GD and WS

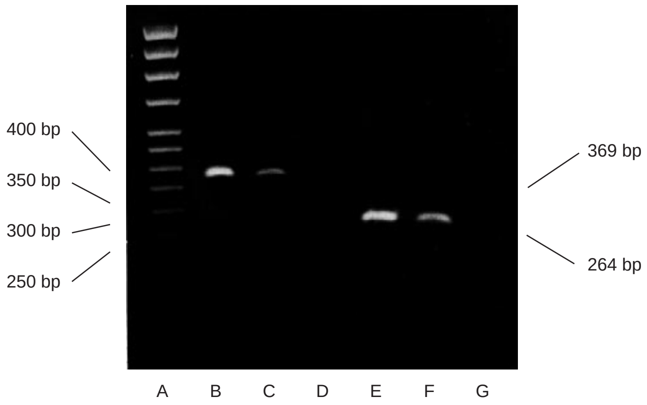

**Figure 3** Allele-specific DNA amplifications in patient WG and BHM. 200 ng of DNA was used in the 35 cycles of PCR in each reaction. The PCR bands of mutant *p53* (369 bp) were shown in both relapse (lane B) and presentation (lane C) samples of patient (WG) and the PCR bands of mutant *p53* (264 bp) were shown in both relapse (lane E) and presentation (lane F) samples of patient (BHM), although the bands in presentations were much weaker than those in relapse, and the mutant bands were confirmed by direct sequencing. No bands were amplified in a further patient (SJ) as a negative control (lane D and lane G). Lane 1 was 50 bp ladder as marker relapse are shown in the Table 1. The mutations of the *p53* gene in ALL occurred at exon 5 in two cases (BHM, WN) and at exon 6 in two cases (IR, WG); in AML the mutations of *p53* occurred at exon 7 in two cases (SR, WS) and at exon 8 in one case (GD). All of these mutations were missense mutations and were confirmed by sequencing with reverse primers. Identical mutations at codon 175 (G→A) (Wada et al, 1993), 193 (Fenaux et al, 1992b; Baldini et al, 1994), 248 (Abo et al, 1993; Trecca et al, 1994) and 273 (Gaidano et al, 1991; Fenaux et al, 1992*a*; 1992*b*; Nishimura et al, 1995) have been previously reported in leukaemia, but the mutations at codons 175 (C→T), 209 and 241 have only been previously reported in solid tumours.

### Analysis of *p53* mutations in presentation samples of patients with mutation at relapse

Part of the purpose of this study was to determine if mutations detected at relapse of acute leukaemia were present in presentation samples. Cryopreserved samples from presentation material were available for analysis in three (BHM, IR and WG) of the four ALL cases and in two (GD and WS) of the three AML cases. The presence of *p53* mutations at presentation was initially studied by direct sequencing of the PCR-amplified exon mutated in the relapse sample. The results for the ALL patients are shown in Figure 1, the identical mutation was detected at presentation in one sample (IR), however as can be seen the mutation was present in a smaller proportion of cells and the majority expressed wild-type sequences. No mutation was detected at presentation in BHM or WG. In both AML patients, the identical mutation was present in the presentation samples using direct sequencing as shown in Figure 2.

### Presence of mutant *p53* at presentation by allele-specific gene amplification

The reported sensitivity of automated direct sequencing or SSCP to detect a mutated population in the presence of wild-type

*© Cancer Research Campaign 1999*

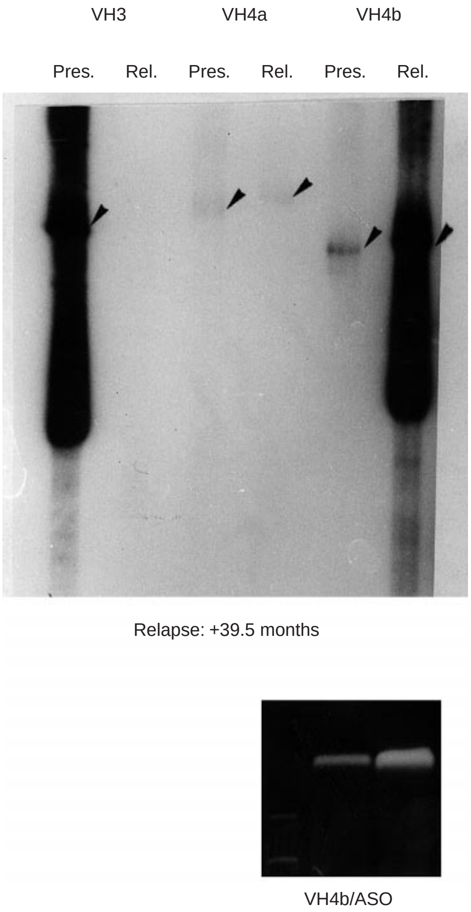

**Figure 4** Clonality of the B-cells was analysed in the presentation (Pros.) and relapse (Rel.) material of BHM by IgH fingerprinting test with combination of primers JH and VH3, or VH4a or VH4b. A major VH3 clone and minor VH4a and VH4b were detected in the presentation (black arrows). However, a major VH4b clone and minor VH4a clone, but not VH3 clone were detected at relapse. Amplification of presentation and relapse DNA by using VH4b (sense primer) and allele-specific oligonucleotides (antisense primer) clearly indicated the identity of the VH4b clone by ethidium bromide gel electrophoresis as shown in the bottom of the figure (left lane: DNA marker, middle lane: presentation sample, right lane: relapse sample)

sequences is only about 10% (Gaidano et al, 1991; Murakami et al, 1991; Larder et al, 1993). As a consequence the failure to detect *p53* mutations in presentation samples of patients BHM and WG might reflect a lack of sensitivity of direct sequencing to detect the presence of a minor clone (< 10% of cells) carrying *p53* mutations. We proceeded to use allele-specific PCR, a far more sensitive method capable of detecting mutated cells with a sensitivity of at least 1 in $10^4$ (Wada et al, 1994), to re-analyse the presentation sample in patients WG and BHM. As shown in Figure 3, the allele-specific oligonucleotides amplified a PCR product with the predicted size (264 bp and 369 bp for BHM and WG, respectively) in both the relapsed and the presentation samples, although the bands in the presentation samples were weaker than those at relapse. The percentage of blasts in the relapsed and presentation samples in both these cases was > 90%. The bands in the presentation samples were purified and sequenced, the sequence result confirming that those were mutated from the gene *p53*. No positive signals were observed using the same primers with DNA from a negative control sample (SJ), in which we had previously established that codon 175 and codon 209 were normal.

### Clonality of the B-cells in ALL patient BHM

As part of a separate study (Papaioannou et al, 1997), clonality of the B cells from patient BHM were further investigated by IgH FR1 fingerprinting. At presentation a major VH3 clone was detected with minor VH4a and VH4b being present. However, at relapse (39 months later) the VH4b clone almost entirely replaced the VH3, which was undetectable as shown in Figure 4.

### DISCUSSION

*p53* mutations are frequently observed in solid tumours, and in a number of haematological malignancies including chronic myeloid leukaemia and follicular lymphoma where the acquisition of *p53* mutation is associated with disease progression and transformation to high grade disease (Ahuja et al, 1989; Lo Coco et al, 1993; Sander et al, 1993). In acute leukaemia, however, the incidence is low, thus Fenaux et al (1992a; 1992b) reported that only one of 50 cases of pre-B ALL and eight out of 112 cases of AML had a demonstrable *p53* mutation. Likewise, Wada et al (1993) found an incidence of *p53* mutations of only 2–3% in a large series of patients with pre-B ALL. Indeed the low incidence of *p53* mutation in childhood ALL has been put forward as an explanation for the high degree of chemosensitivity of this tumour (Lowe et al, 1993).

As with current treatment protocols, approximately 50% of adult patients with AML or ALL will relapse after achieving CR and as *p53* mutations might be expected to be associated with a poor response to therapy we decided to analyse samples from relapsed patients for the presence of *p53* mutations in exons 5–9 in order to establish whether the biological significance of *p53* mutation in the evolution of acute leukaemia had been underestimated by analysing presentation samples. The majority of patients studied had been entered into UK Medical Research Council trials for the treatment of AML or ALL and all had received aggressive chemotherapy and we took care to exclude any patients with secondary AML or antecedent myelodysplasia who might be more likely to have *p53* mutations (Sugimoto et al, 1993). SSCP is the most commonly used method for detection of *p53* mutations with a sensitivity for detecting mutated cells in the presence of wild-type sequence of about 10% (Gaidano et al, 1991; Murakami et al, 1991; Larder et al, 1993). However SSCP may also miss *p53* mutations due to lack of conformational change and therefore the use of SSCP as a screening assay may underestimate the frequency of mutations (Martincic and Whitlock, 1996). For this study we therefore decided to directly sequence exon 5–9 of all relapsed samples. We found a higher incidence of *p53* mutations in relapsed samples than has been reported for ALL at presentation. Overall, four out of 14 ALL patients (28.6%) had a *p53* mutation at relapse which compared with just three out of 41 AML patients (7.3%), an incidence comparable to that reported for AML at presentation. Our results in ALL are comparable to those reported for relapsed T-ALL by Diccianni et al (1994). All of the ALL mutations were missense mutations, two of them in codon 175, which is a frequent site of *p53* mutation. In three of the four ALL patients presentation samples were available for analysis, which confirmed the presence of the mutation by direct sequencing in one case. This patient had B-ALL, which is known to be associated with a higher incidence

© Cancer Research Campaign 1999

of *p53* mutation than other types of ALL (Gaidano et al, 1991). By direct sequencing, presentation samples from the other two ALL patients showed no detectable mutation but using allele-specific PCR with appropriate controls, a band was amplified at both presentation and relapse in both cases. It is interesting that one of those patients was investigated by IgH fingerprinting and a VH4 rearrangement present in only a minor subclone at presentation was the predominant clone at relapse; one might speculate that the VH4 subclone also harboured the *p53* mutation present at relapse. These observations suggest that in ALL, cells carrying *p53* mutations are present early in the natural history of the disease in some patients but our studies only allow a crude estimate of the size of this population (0.01–10%). Resistance to chemotherapy-induced apoptosis may confer a survival advantage to a *p53*-mutated subclone which by clonal expansion could mediate relapse. This may be of biological importance in ALL where previous studies have demonstrated oligoclonality in 25–30% of cases at diagnosis (Beishuizen et al, 1991; Coyle et al, 1996). We did not find a higher incidence of *p53* mutations at relapse of AML. However, this does not mean that *p53*-mutated subclones do not mediate relapse in AML as one of the patients studied by Wada et al (1994) had AML. The kinetics of a relapse will depend upon the size of the mutated clone at presentation and other biological variables. Our results may indicate that *p53* mutations are a determinant of outcome in both adults and children with ALL being implicated in both primary treatment failure as well as relapse in a proportion of patients (Diccianni et al, 1994; Marks et al, 1996). It would be of interest to confirm these results on a larger series of patients in a prospective manner. The development of new techniques to detect *p53* mutations in a small clone of leukaemic cells at presentation, which are not dependent upon prior knowledge of the sequence, may be of value in identifying patients with a high risk of disease recurrence.

### ACKNOWLEDGEMENT

This work was supported by grants from Leukaemia Research Fund to NHR and LF.

### REFERENCES

Abo J, Inokuchi K, Dan K and Nomura T (1993) p53 and N-ras mutations in two new leukemia cell lines established from a patient with multilineage CD7-positive acute leukemia. *Blood* **82**: 2829–2836

Ahuja H, Bar-Eli M, Advani SH, Benchimol S and Cline MJ (1989) Alterations in the *p53* gene and the clonal evolution of the blast crisis of chronic myelocytic leukemia. *Proc Natl Acad Sci USA* **86**: 6783–6787

Baldini L, Stefano-Fracciolla N, Cro LM, Trecca D, Romitti L, Polli E, Maiolo AT and Neri A (1994) Frequent *p53* gene involvement in splenic B-cell leukaemia/lymphomas of possible marginal zone origin. *Blood* **84**: 270–278

Beishuizen A, Hahlen K, Hagemeijer A, Verhoeven MAJ, Hooijkaas H, Adriaansen HJ, Wolverstettero ILM, Vanwering ER and Vandongen JJM (1991) Multiple rearranged immunoglobulin genes in childhood acute lymphoblastic leukemia of precursor B-cell origin. *Leukemia* **5**: 657–667

Ben-Yehuda D, Krichevsky S, Caspi O et al (1996) Microsatellite instability and *p53* mutations in therapy-related leukemia suggest mutator phenotype. *Blood* **88**: 3022–3026

Chim JCS, Coyle LA, Yaxley JC, Cole-Sinclair MF, Cannell PK, Hoffbrand VA and Foroni L (1996) The use of IgH fingerprinting and ASO-dependent PCR for the investigation of residual disease (MRD) in ALL. *Br J Haematol* **92**: 104–115

Coyle LA, Papaioannou M, Yaxley JS, Chim JS, Attard M, Hoffbrand AV and Foroni L (1996) Molecular analysis of the leukaemic B-cell in adult and childhood acute lymphoblastic leukaemia. *Br J Haematol* **94**: 685–693

Diccianni MB, Yu J, Hsiao M, Mukherjee S, Shao L-E and Yu AL (1994) Clinical significance of *p53* mutations in relapsed T-cell acute lymphoblastic leukemia. *Blood* **84**: 3105–3112

Fenaux P, Jonveaux P, Quiquandon I, Lai JL, Pignon JM, Loucheux-Lefebvre MH, Bauters F and Kerckaert JP (1991) *p53* gene mutations in myeloid leukemia with 17p monosomy. *Blood* **78**: 1652–1657

Fenaux P, Preudhomme C, Quiquandon I, Jonveaux P, Lai JL, Vanrumbeke M, Loucheux-Lefebvre MH, Bauters F, Berger R and Kerchaert JP (1992*a*) Mutations of the *p53* gene in acute myeloid leukaemia. *Br J Haematol* **80**: 178–183

Fenaux P, Jonveaux P, Quiquandon I, Preudhomme C, Lai JL, Vanrumbeke M, Loucheux-Lefebvre MH, Bauters F, Berger R and Kerckaert JP (1992*b*) Mutations of the *p53* gene in B-cell lymphoblastic acute leukemia: a report on 60 cases. *Leukemia* **6**: 42–46

Gaidano G, Ballerini P, Gong JZ, Inghirami G, Neri A, Newcomb EW, Magrath IT, Knowles DM and Dalla-Favera Rl (1991) *p53* mutation in human lymphoid malignancies associated with Burkitt lymphoma and chronic lymphocytic leukemia. *Proc Natl Acad Sci USA* **88**: 5413–5417

Hsiao MH, Yu AL, Yeargin J, Ku D and Haas M (1994) Nonhereditary *p53* mutations in T-cell acute lymphoblastic leukemia are associated with the relapse phase. *Blood* **83**: 2922–2930

Hu G, Zhang W and Deisseroth AB (1992) *p53* gene mutations in acute myelogenous leukaemia. *Br J Haematol* **81**: 489–494

Jonveaux P and Berger R (1991) Infrequent mutations in the *p53* gene in primary human T-cell acute lymphoblastic leukemia. *Leukemia* **5**: 839–840

Lane DP and Benchimol S (1990) *p53*: oncogene or anti-oncogene? *Genes & Development* **4**: 1–8

Larder BA, Kohli A, Kellam P, Kemp SD, Kronick M and Henfrey RD (1993) Quantitative detection of HIV-1 drug resistance mutations by automated DNA sequencing. *Nature* **365**: 671–673

Lo Coco F, Gaidano G, Louie DC, Offit K, Chaganti RSK and Dalla-Favera R (1993) *p53* mutations are associated with histologic transformation of follicular lymphoma. *Blood* **82**: 2289–2295

Lowe SW, Ruley HE, Jacks T and Housman DE (1993) *p53*-dependent apoptosis modulates the cytotoxicity of anticancer agents. *Cell* **74**: 957–967

Marks DI, Kurz BW, Link MP, Ng E, Shuster JJ, Lauer SJ, Brodsky I and Haines DS (1996) High incidence of potential *p53* inactivation in poor outcome childhood acute lymphoblastic leukemia at diagnosis. *Blood* **87**: 1155–1161

Martincic D and Whitlock JA (1996) Improved detection of *p53* point mutations by dideoxyfingerprinting (ddF). *Oncogene* **13**: 2039–2044

Mori N, Wada M, Yokota J, Terada M, Okada M, Teramura M, Masuda M, Hoshino S, Motoji T, Oshimi K and Mizoguchi H (1992) Mutations of the *p53* tumour suppressor gene in haematologic neoplasms. *Br J Haematol* **81**: 235–240

Murakami Y, Hayashi K and Sekiya T (1991) Detection of aberrations of *p53* alleles and the gene transcript in human tumor cell lines by single-strand conformation polymorphism analysis. *Cancer Res* **51**: 3356–3361

Nakai H, Misawa SH, Toguchida J, Yandell DW and Ishizaki K (1992) Frequent *p53* gene mutations in blast crisis of chronic myelogenous leukemia, especially in myeloid crisis harboring loss of a chromosome. *Cancer Res* **52**: 6588–6593

Nishimura S, Asou N, Suzushima H, Okubo T, Fujimoto T, Osato M, Yamasaki H, Lisha L and Takatsuki K (1995) *p53* gene mutation and loss of heterozygosity are associated with increased risk of disease progression in adult T-cell leukemia. *Leukemia* **9**: 598–604

Papaioannou M, Yaxley JC, Hoffbrand AV and Foroni L (1997) Oligoclonality in acute lymphoblastic leukaemia (ALL): emergence of minor clones rather than change of clonality at relapse in ALL. *Br J Haematol* **97** (Suppl 1): 202a

Sander CA, Yano T, Clark HM, Harris C, Longo DL, Jaffe ES and Raffeld M (1993) *p53* mutation is associated with progression in follicular lymphomas. *Blood* **82**: 1994–2004

Slingerland JM, Minden MD and Benchimol S (1991) Mutation of the *p53* gene in human acute myelogenous leukemia. *Blood* **77**: 1500–1507

Sugimoto K, Hirano N, Toyoshima H, Cluiba S, Mano H, Takaku F, Yazaki Y and Hirai H (1993) Mutations of the *p53* gene in myelodysplastic syndrome (MDS) and MDS-derived leukemia. *Blood* **81**: 3022–3026

Sugimoto K, Toyoshima H, Sakai R, Miyagawa K, Hagiwara K, Ishikawa F, Takaku F, Yazaki Y and Hirai H (1992) Frequent mutations in the *p53* gene in human myeloid leukemia cell lines. *Blood* **79**: 2378–2383

Trecca D, Longo L, Biondi A, Cro L, Calori R, Grigani F, Maiolo AT, Pelicci PG and Neri A (1994) Analysis of *p53* gene mutations in acute myeloid leukemia. *Am J Hematol* **46**: 304–309

Vogelstein B (1990) A deadly inheritance. *Nature* **348**: 681–682

© Cancer Research Campaign 1999

Wada H, Asada M, Nakazawa S, Itoh H, Kobayashi Y, Inoue T, Fukumuro K, Chan LC, Sugita K, Hanada R, Akuta N, Kobayashi N and Mizutani S (1994) Clonal expansion of *p53* mutant cells in leukemia progression in vitro. *Leukemia* **8**: 53–59

Wada M, Bartram CR, Nakamyra H, Hachiyam Chen DL, Borenstein J, Hansen-Hagge TE, Ludwig WD, Reiter A, Mizoguchi H and Koeffler HP (1993) Analysis of *p53* mutations in large series of lymphoid hematological malignancies of childhood. *Blood* **82**: 3163–3169

Wattel E, Preudhomme C, Hecquet B, Vanrumbeke M, Quesnel B, Dervite I, Morel P and Fenaux P (1994) *p53* mutations are associated with resistance to chemotherapy and short survival in hematologic malignancies. *Blood* **84**: 3148–3157

© Cancer Research Campaign 1999
